# Combined analysis of chromosomal instabilities and gene expression for colon cancer progression inference

**DOI:** 10.1186/2043-9113-4-2

**Published:** 2014-01-24

**Authors:** Claudia Cava, Italo Zoppis, Manuela Gariboldi, Isabella Castiglioni, Giancarlo Mauri, Marco Antoniotti

**Affiliations:** 1 Institute of Molecular Bioimaging and Physiology of the National Research Council (IBFM-CNR), LITA Building - Via F.lli Cervi 93, 20090 Segrate (MI), Italy; 2 Dipartimento di Informatica, Sistemistica e Comunicazione, Università degli Studi di Milano Bicocca, Viale Sarca 336, U14, 20126 Milan, Italy; 3 Department of Experimental Oncology, Fondazione IRCCS Istituto Nazionale dei Tumori, Milan, Italy; 4 Molecular Genetics of Cancer, FIRC Institute of Molecular Oncology Foundation, Milan, Italy

**Keywords:** Copy number alteration, Dissimilarity representation, Colorectal cancer, Support vector machine

## Abstract

**Background:**

Copy number alterations (CNAs) represent an important component of genetic variations. Such alterations are related with certain type of cancer including those of the pancreas, colon, and breast, among others. CNAs have been used as biomarkers for cancer prognosis in multiple studies, but few works report on the relation of CNAs with the disease progression. Moreover, most studies do not consider the following two important issues. (I) The identification of CNAs in genes which are responsible for expression regulation is fundamental in order to define genetic events leading to malignant transformation and progression. (II) Most real domains are best described by *structured* data where instances of multiple types are related to each other in complex ways.

**Results:**

Our main interest is to check whether the colorectal cancer (CRC) progression inference benefits when considering both (I) the expression levels of genes with CNAs, and (II) relationships (i.e. dissimilarities) between patients due to expression level differences of the altered genes. We first evaluate the accuracy performance of a state-of-the-art inference method (support vector machine) when subjects are represented only through sets of available attribute values (i.e. gene expression level). Then we check whether the inference accuracy improves, when explicitly exploiting the information mentioned above. Our results suggest that the CRC progression inference improves when the combined data (i.e. CNA and expression level) and the considered dissimilarity measures are applied.

**Conclusions:**

Through our approach, classification is intuitively appealing and can be conveniently obtained in the resulting dissimilarity spaces. Different public datasets from *Gene Expression Omnibus* (GEO) were used to validate the results.

## Background

Colorectal cancer (CRC) is the third most common cancer worldwide. The life expectancy of individuals with CRC is mainly dependent on the clinical stage which may characterize the disease according e.g., to the following tumor progression (Duke’s stage classification) system [1]. 

**Figure 1:**
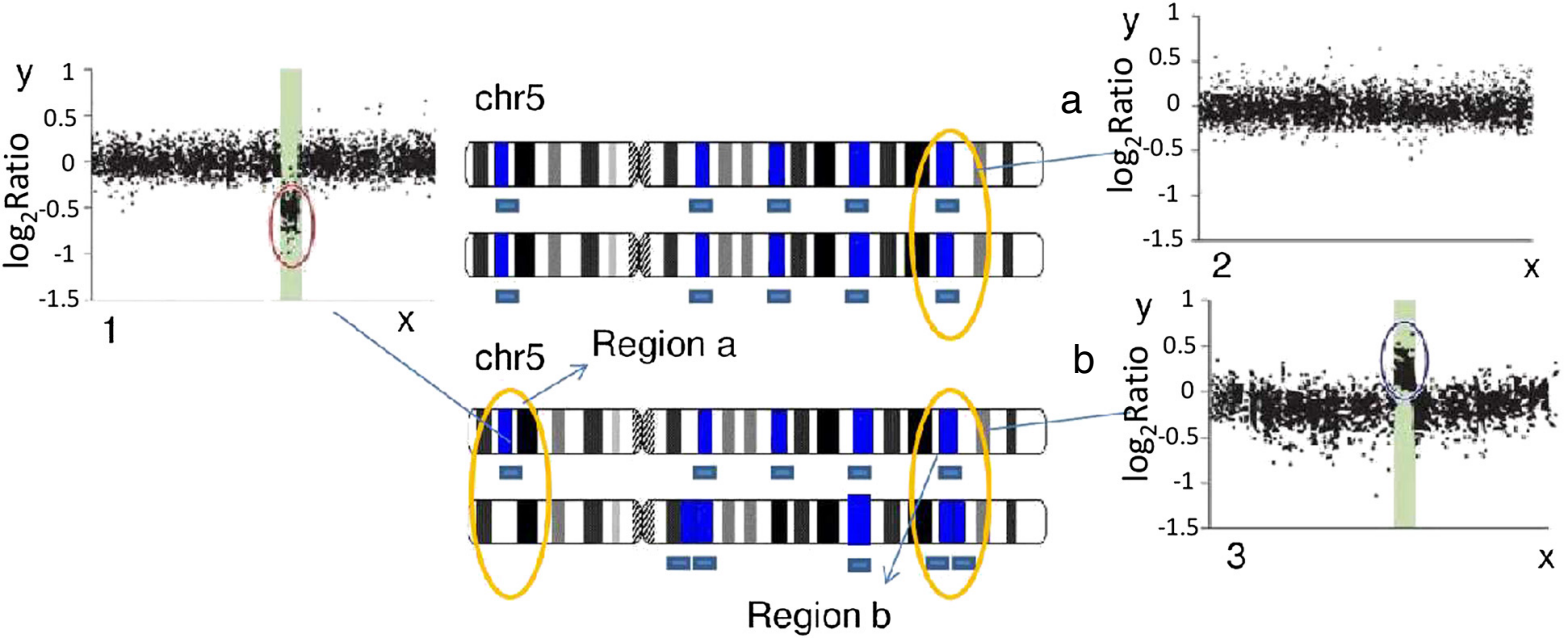
**Copy–number alterations.** Intensities of single-nucleotide polymorphisms (SNPs) are plotted (black dots). x-Axis: chromosomal positions. y-Axis: log intensity. *Normal situation*: DNA regions (colored bars) are present as two diploid copies on chromosome **a**. SNP’s intensity values is close to 0 (plot 2). *Loss region*: intensity decreases (plot 1) due to *region-a* deletion on chromosome **b**. *Gain region* – intensity increases (plot 3) due to *region-b* duplication on chromosome **b**.

• **Stage I**: CRC is only in the innermost lining of the colon or rectum or slightly growing into the muscle layer;

• **Stage II**: CRCs are extended through the muscular wall of the colon but do not affect the lymph nodes;

• **Stage III**: CRCs have spread outside the colon to one or more lymph;

• **Stage IV**: CRCs have spread outside the colon to other parts of the body commonly the liver or the lungs;

Stage-I patients have a 5-year survival rate of approximately 93% which decreases to approximately 80% for patients with stage II, 60% for patients with stage III and, 8% for stage IV [2]. The development and progression of CRC (as for most other solid cancers) is a multi-step process also leading to the accumulation of chromosomal instability (CIN) that occurs over the lifetime of a tumor. Three major forms of genetic instability in CRC have been described: microsatellite instability (MIN), epigenetic changes (as DNA methylation) and chromosomal instability which leads to gains and losses of chromosomal segments [3-5]. CINs include DNA *copy number alterations* (CNAs), i.e., regions of aberrantly increased or decreased DNA (see Figure 1). Such alterations ultimately leads to malignant transformation and progression [6].

The need to better understand tumor genesis and its relationship with CNAs has led many studies to attack the problem from different prospectives; many of which have been enabled recently by an increasing and multifarious set of tools and techniques in cancer research [7]. For example, Leslie et al. [8] investigated on the aberration frequency of the colorectal neoplasia providing significant evidence of both (aberration) gain at chromosomes 20 q, 13 q, 7 p, 8 q and (aberration) loss at 18 q, 17 p, 8 p.

Differently, Bomme et al. [9] showed the relationship between tumor progression and metastases with CNA positions over the chromosomes. They observed one of the earliest gathered genetic abnormalities related to chromosome 7 amplification during the colorectal cancer (CRC) progression. Moreover, Ghadimi et al. [10] reported the potential role of chromosome 8 q amplification for the development of lymph node metastases.

Most studies concerning CNAs investigate the use of aberrations as biomarkers for cancer prognosis (e.g., [11,12]), but few works report on the relationship of CNAs with the disease progression [13-17]. Moreover, most of these studies do not consider the following two important issues. 

• The identification of CNAs in genes which are responsible for expression regulation is fundamental in order to define key genetic events leading to malignant transformation and disease progression. By combining gene expression and copy number data these regulators can be revealed. Only a limited number of studies apply this approach, for instance in breast cancer prognosis [18,19]. Other authors used high resolution oligonucleotide comparative genomic hybridization arrays, and by matching gene expression array data showed correlation between DNA copy number alteration and mRNA levels [20].

• Most real domains are best described by *structured* data where instances of multiple types are related to each other in complex ways. For example, scientific papers are related through citations and authors, web pages are interconnected by hyperlinks, telephone accounts are linked by calls. Nevertheless, in clinical investigation, classification is generally obtained assuming that *case* or *control* subjects are independent and identically distributed (IID). Numerous algorithms have been designed to work on such (as we will call in this paper) “standard approach”, where instances (e.g. patients) can be represented as fixed-length vectors of attribute values (see [21] for a survey). Actually, the CNAs within a patient group might be related each other, and this property in turn may change when the relationship is defined over different groups. Moreover, when the relationships are addressed through dissimilarities [22], the resulting patient representation (i.e., *dissimilarity representation*) is intuitively appealing and is supported by the fact that classification (and clustering) methods can be suitably applied in the resulting “dissimilarity space” [22].

The main issue of our investigation is to check whether the accuracy of the CRC progression inference benefits when considering the following types of information. 

• Expression levels of altered genes, and

• relationships (i.e., dissimilarities) among patients due to expression level differences of the altered genes.

In the first case only the expression level of altered genes is used with standard inference mechanisms (here, we call this approach “*combined approach*”, shortly COMB). In the second case we define dissimilarities among patients due to differences among the COMB data associated to each subject, and evaluate the “inference accuracy” when using this new type of representation; we call this approach “*relational approach*” (shortly RA). Specifically, our inference is based on “control vs. case” classification tasks. In other words, given a patient *x*, whose stage is, e.g., stage(*x*), we evaluate the ability of an inference mechanism to classify that patient either in the same stage (i.e., stage(*x*)) or in an advanced stage, say stage^′^>stage(*x*). Our evaluation (provided through comparisons) is empirical: we first observe the accuracy performance of a state-of-the-art inference method (for instance *Support Vector Machine*) to forecast the CRC stage progression when patients are represented through the set of available attribute values only given by the gene expression levels. As mentioned above, we call this approach standard (shortly SA) since this reflects a typical way of representing IID subjects. Then we check whether the inference accuracy improves when explicitly exploiting both the information provided respectively through COMB and RA.

In order to obtain the expression level of genes with CNAs, we first identify differentially expressed genes by evaluating their expression levels from different datasets (see below in the text). Similarly, altered genes (i.e., genes with amplification or deletion) are identified by analyzing their CNAs from different datasets. Then, by considering the results of both the gene expression analysis and the CNA analysis, we obtain up-regulated genes with CNA gains and down-regulated genes with CNA losses.

Moreover, in order to quantify relationships between patients which can express, as stated above, the CRC progression, we define a *dissimilarity* over both an “advanced-stage” patient group and a specific “representative” base group, e.g. patients with the lowest stage (which we will refer to as “prototype” group). As previously mentioned, the considered dissimilarities quantify, by construction, subject differences due to different expression levels of altered genes (as obtained via the previous analysis) belonging to each subject.

While in a SA, subjects are discriminated on their own set of attribute values, in the *dissimilarity-based* classification we consider, we employ pairwise comparisons (between patients), i.e., a *N*×*N* dissimilarities matrix *D*(*T*,*P*). Each entry of *D*(*T*,*P*) is a dissimilarity value computed between pairs of patients that is, each patient *x* within the group *T* is represented by a vector of *dissimilaritiesD*(*x*,*P*) to patients of a representative (prototype) group *P*.

Dissimilarities have been used in *pattern recognition* for many years, leading to many different known algorithms and important questions. For example, the idea of “template matching” is based on dissimilarities: objects are given the same class label if their difference is sufficiently small [23]. This is identical to the nearest neighbor rule used in vector spaces [21]. Also many procedures for cluster analysis make use of dissimilarities instead of the standard feature space representation [24]. A use of dissimilarity measures to reconstruct dynamic temporal models of biological processes can be found in [25] A detailed description, providing mathematical foundation, designed procedures, and real world examples for building pattern recognition systems based on dissimilarity representation may also be found in [22].

## Materials and methods

The description of the material and methods we used in our study can be conveniently organized according to the type of analysis conducted, as listed hereafter. 

1. Gene expression analysis.

2. Copy number analysis.

3. Combined gene expression and CNA analysis.

4. Dissimilarity-based representation.

5. Inference procedure.

6. Statistical evaluations.

Table 1 shows the classification tasks that we defined as the “drivers” of our study.

**Table 1:** Inference tasks

	***Control group***	**VS**	***Case group***
*Task 1*	Stage II		Stage III
*Task 2*	Stage II		Stage IV
*Task 3*	Stage III		Stage IV

I.e., the disease progression inference is based on *control vs. case* classification tasks. Please note that we used as control group the patients with the lowest stage in the considered tasks (e.g., stage II, when considering stage-II vs. stage-III). In this work all the control groups (i.e., tumor progression negatives) are labeled by 0, while the remaining (i.e., positive) are labeled by 1. Moreover, we point out that the *dissimilarity-based representation* is based on the work of Pekalska et al. [22] and is adapted here to conclusively provide the results. For this reason, we will detail the description (i.e. formulation) of this representation.

### Gene expression analysis

In this phase, differentially expressed genes (up or down–regulated) were selected by evaluating their expression levels on different datasets [26,27]. For this, we used two public CRC microarray data from Gene Expression Omnibus (GEO) [28]: GSE27854 and GSE17536. From the first dataset three groups of patients were selected: 41 patients with stage II, 35 patients with stage III, and 23 with stage IV. Similarly, from the second dataset the following three groups of patients were selected: 57 patients with stage II, 57 with stage III, and 39 with stage IV.

**Figure 2:**
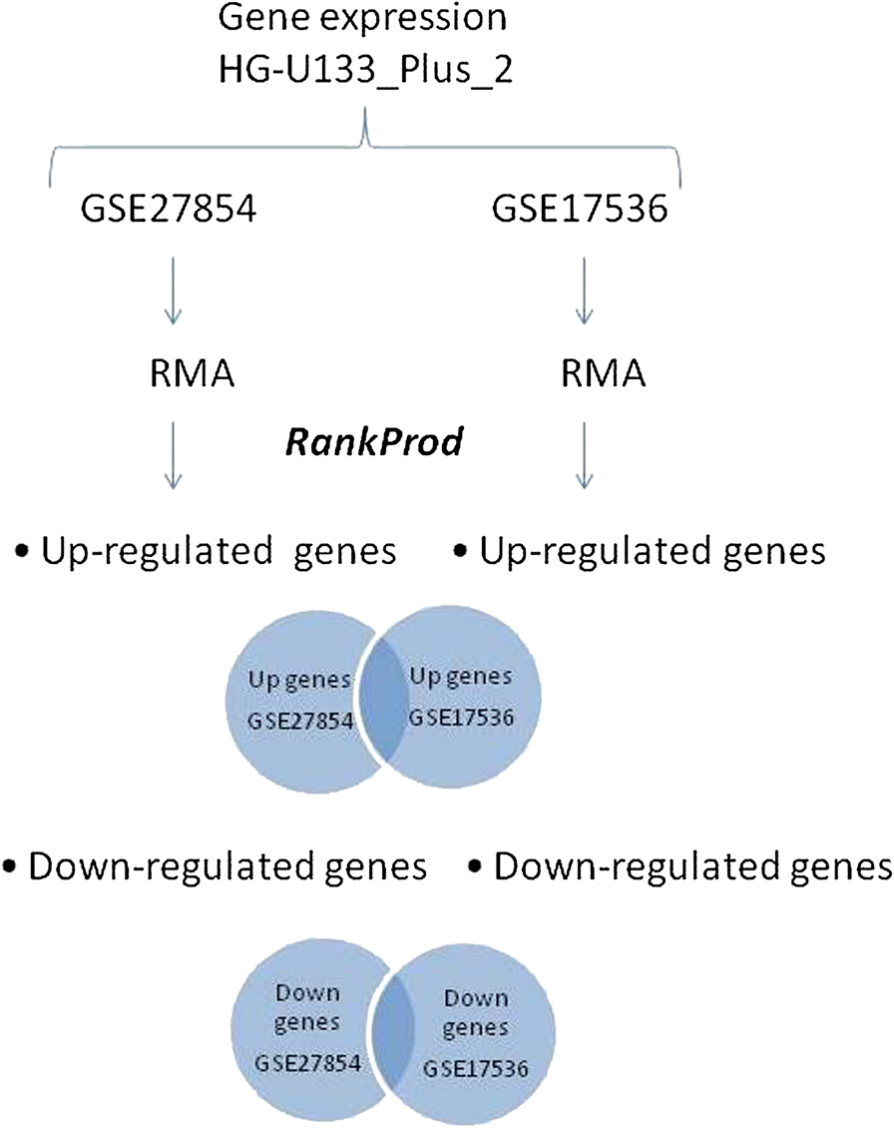
**Gene expression analysis.** Expression values is obtained in both datasets. RankProd is applied for identifying differentially expressed (up/down–regulated) probes. Up/down expressed genes were identified by submitting IDs probes to the Netaffx tool.

Given any dataset and a specific task in Table 1, we say that a gene is *differentially expressed for that dataset* if it is up- (down-) expressed in the highest stage patients in comparison to the lowest stage patients of that dataset. When a gene is differentially expressed in both datasets (i.e., GSE27854 and GSE17536), we conclusively consider that genes as *differentially expressed* and apply it to the combined data analysis as we will report in the following paragraphs. In other words, we use more than one dataset to give more evidence for a gene to be up/down-regulated. This procedure is summarized as follows (we also represent this analysis in Figure 2): 

• Expression values from Affymetrix Human Genome U133 Plus 2.0 array were calculated for both datasets. For this, we used a robust multi-array average (RMA) [29] method present in the R statistical software. Our aim was to select significant genes based on differential expression between patient stages.

• RankProd [30] was applied for identifying differentially expressed (up/down-regulated) probes based on the estimated percentage of false predictions (pfp). We fixed the significance cut-off using *p*-values by setting the (default) *α* parameter required by the software to 0.01, cfr., [31]. More specifically, the RankProd analysis was used as a first step in both datasets. Thus we obtained DNA probes which are up/down expressed in the highest stage patients in w.r.t. the lowest stage patients.

• Finally, up/down expressed genes were identified by submitting IDs probes (obtained through RankProd) to the Netaffx tool [32].

### Copy number analysis

As in the previous analysis, in this phase we use more than one dataset to obtain more supporting evidence for a gene amplification/deletion. To this aim, we used three public CRC microarray (GEO) data: GSE16125, GSE11417 and GSE27910.

The first dataset was provided by the *Fondazione IRCCS Istituto Nazionale dei Tumori* (INT) and deposited on GEO (GEO16125) [6]. In this dataset, tissue specimens from 53 consecutive sporadic CRCs were obtained from previously untreated patients who underwent surgical resection at INT between 1998 and 2000. 51 DNA samples were hybridized to Affymetrix GeneChipVR Human Mapping 250 K NspI (SNP arrays). Some samples were excluded due to poor quality hybridizations and unknown stage tumor progression. Also, stage-I patients were excluded because of the lack of instances in the considered data. The analyzed samples can be summarized as follow: 10 stage-II patients, 10 stage-III patients and 23 stage-IV patients.

The second dataset was the GEO CRC GSE11417 [33]. Tumor samples and paired normal tissues were hybridized to Affymetrix Mapping 50 K Xba 240 arrays. CNAs for each sample are obtained between pairs of tumors and normal samples. The dataset is composed of 94 patients (42 with lymph node metastasis): 3 patients with stage 1 (Duke system), 46 patients with stage 2, 37 patients with stage 3 and 8 patients with stage 4.

Further analysis was conducted on the GEO CRC GSE27910 [34]. We investigated 122 patients with CRC from Affymetrix DNA Sty array: 18 patients with stage 1, 42 with stage 2, 37 with stage 3 and 25 with stage 4.

**Figure 3:**
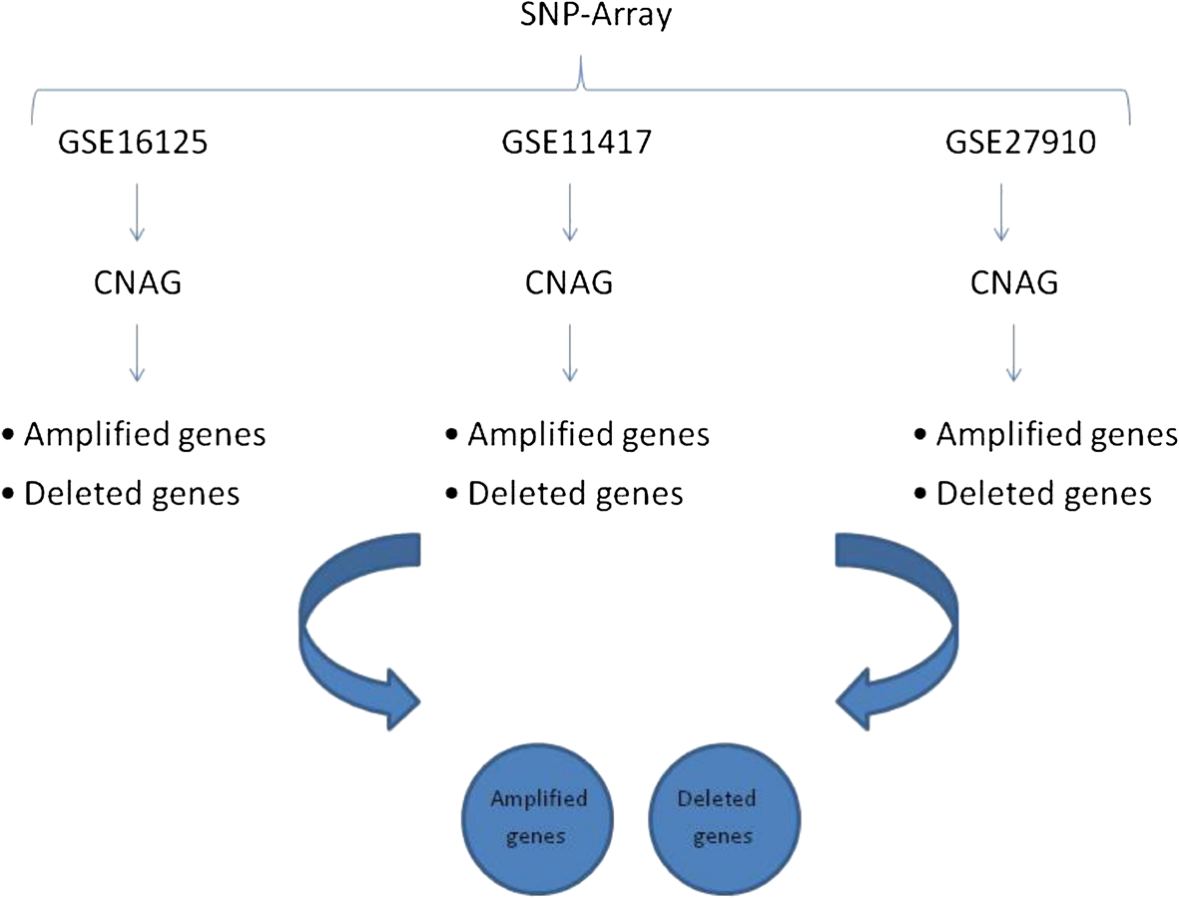
**Copy number analysis.** Amplified and deleted genes are selected with CNAG from each dataset. Common genes are considered altered for combination analysis.

We summarize the CNA analysis procedure (see Figure 3) as follows. 

• For each dataset, we applied CNAG [35] to identify both the sets of amplified and deleted genes.

• Finally, we selected those genes whose alterations were verified on at least two input datasets. Such genes were considered as altered.

### Combination of gene expression levels and copy number alterations

**Figure 4:**
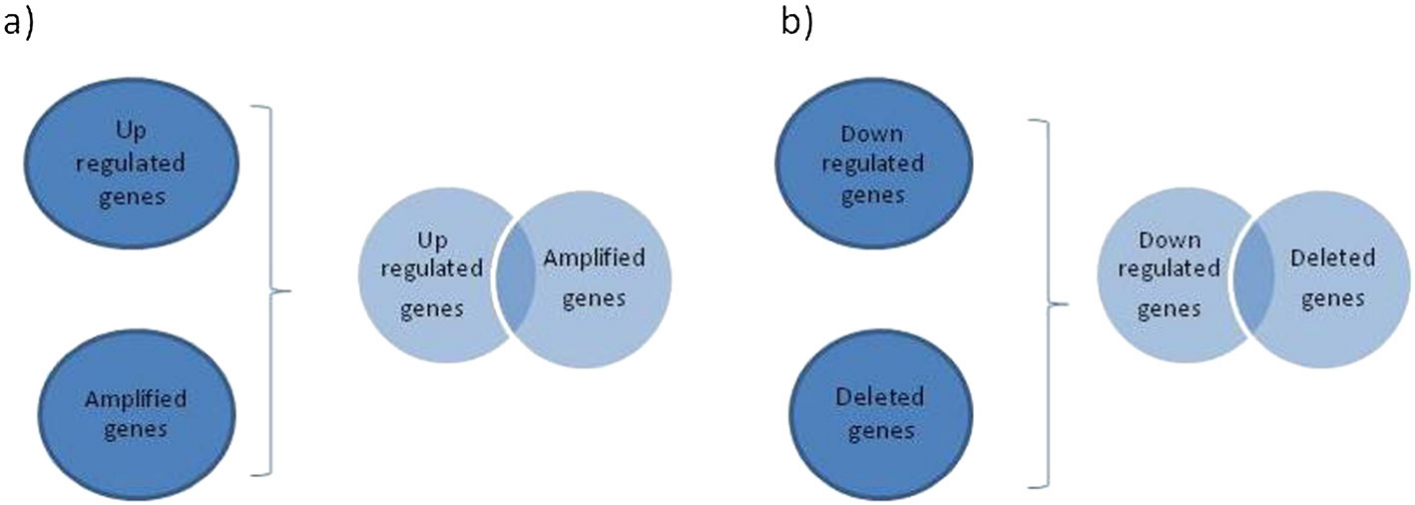
**Combination of gene expression levels and copy number alterations.** Common genes are selected for the inference analysis. **a)** up-regulated and amplified genes **b)** down-regulated and deleted genes.

In this phase, we obtained identification of differentially expressed genes with CNAs gains/losses (see Figure 4).

In particular, by considering the results of the gene expression analysis (i.e., up and down-regulated genes) and the CNA analysis (i.e., amplified and deleted genes), we selected the following genes. 

• Up-regulated genes with CNA gains (by selecting genes common to the set of up–regulated and the set of amplified genes).

• Down-regulated genes with CNA losses (by selecting genes common to the set of down–regulated and the set of deleted genes).

### Dissimilarity-based representation

In the previous sections, we selected differentially expressed genes with CNAs over the chromosomes. Here, we consider relationships among patients: i.e., we define the *dissimilarity representation* among patient.

As noted above, a typical way of representing instances (to be classified) is through the selection of a vector of available attribute values (e.g., gene expression levels). Our goal is to give a *dissimilarity representation* which can express, through a function *D*(*x*,*y*), the dissimilarity between the expression levels of altered genes for the pair of patients *x* and *y*. By extending *D*(*x*,*y*) for all patient pairs, we can construct a dissimilarity matrix whose rows can also be assessed by representing any patient x∈X through the mapping $(X,P)\to R^{n}$ defined as $\varphi (x,P)=\left[D(x,y_{1}),D(x,y_{2}),\ldots ,D(x,y_{n})\right]$, where  and  respectively denote a set of *case/control patients* and a set of *nprototype patients*. Here the difference between  and  reflects the need to discriminate case/control patients in  as compared to a common set of *n* prototype patients in . For instance, this function should be applied to discriminate a stage-III patient $x_{1}\in X$ from a stage-IV patient $x_{2}\in X$, mainly on the basis of the sequences of differences $\varphi (x_{1},P)=\left[D(x_{1},y_{1}),D(x_{1},y_{2}),\ldots ,D(x_{1},y_{n})\right]$ and $\varphi (x_{2},P)=\left[D(x_{2},y_{1}),D(x_{2},y_{2}),\ldots ,D(x_{2},y_{n})\right]$ concerning respectively, (i) dissimilarities between the patient $x_{1}\in X$ from the other prototype patients $y_{i}\in P$, and (ii) dissimilarity between the patient $x_{2}\in X$ from the other prototype patients $y_{i}\in P$. The choice of a correct prototype set can be critical in this approach, and may change the results being investigated. Here we do not study the best possible prototype, instead we employ the group with the lowest stage. As our data does not provide a sufficient number of stage-I patients, we use the stage-II patients as the *prototype set*. Another critical aspect of this representation concerns the definition of a well-discriminating dissimilarity function *D* for a non-trivial learning problem. The following ordinary distances (from the R bioDistance package [36]) are considered: *Euclidean distance*, *Manhattan distance*, *Kendall’s τ-distances* and *Kullback-Leibler distance*.

**Figure 5:**
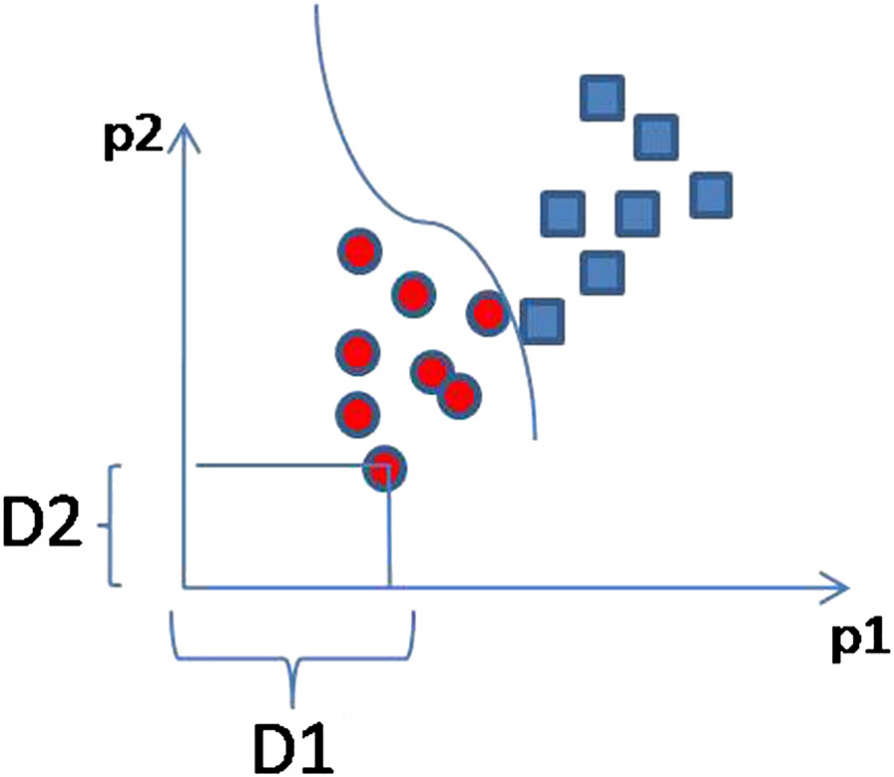
**Classification in *dissimilarity space *.** Patients (points) are discriminated on the basis of their distances (*D*1 and *D*2) to *prototype* patients p1 and p2.

Using this formulation, classification (or clustering) algorithms can be applied to the resulting *dissimilarity space* ($R^{n}$), in which each dimension expresses a dissimilarity with a prototype patient. Figure 5 gives a simple example of the representation for the Euclidean plane (*n*=2).

### Inference procedure and validation datasets

**Figure 6:**
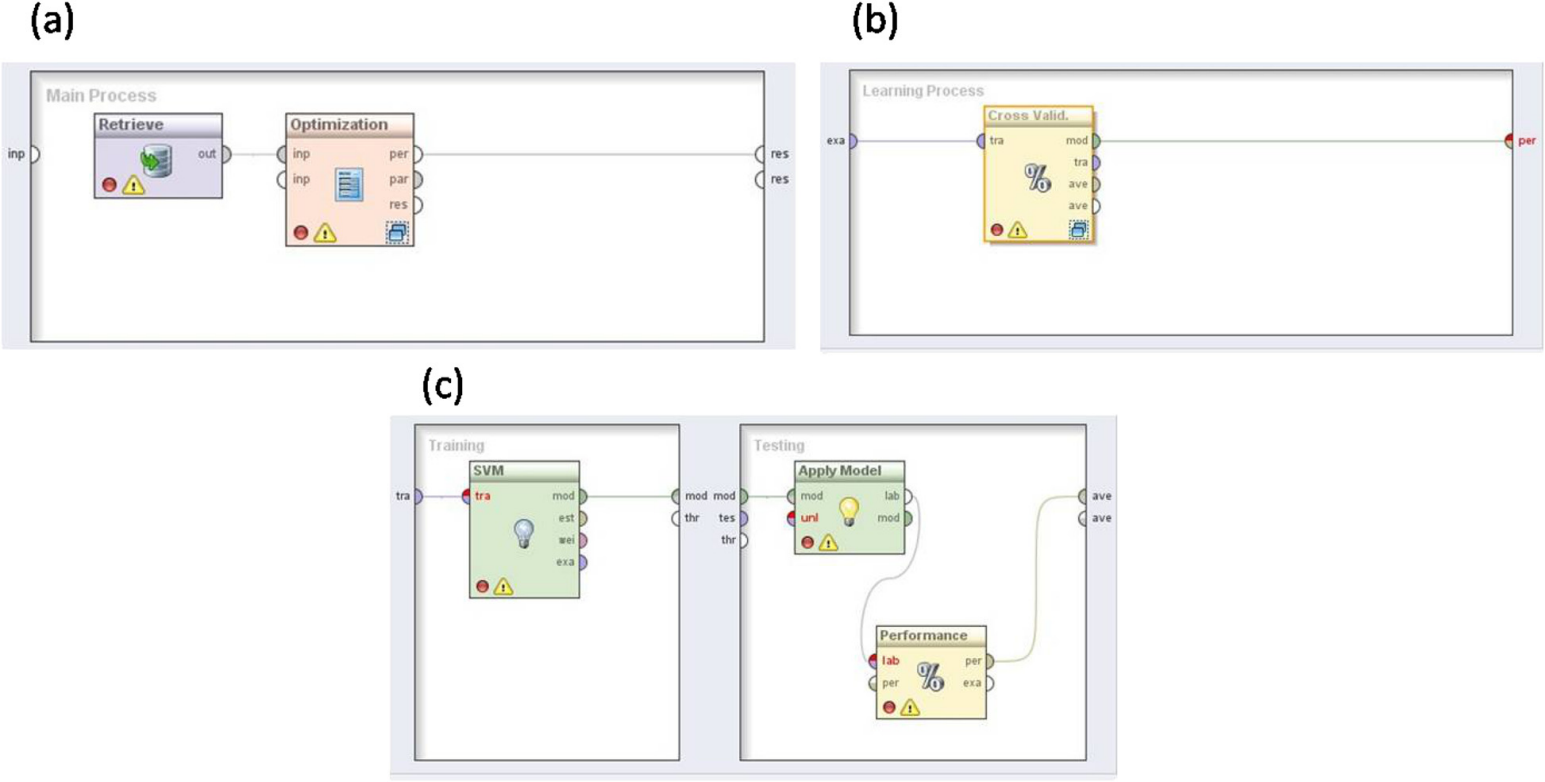
**Rapid miner workflow.** Each operator (RM block) receives an input and delivers an output to the forward operator. **(a)** Optimization iteratively cycles its nested operators (i.e. cross-validation) and change their parameters to optimize the performance of the learning scheme. **(b)** Cross-Validation operator encapsulates a 10-fold cross-validation process. **(c)** The first inner operator (SVM) realizes the SVM Training phase. The second inner operator (Apply Model) tests the trained SVM with new examples.

In order to construct the disease progression inference on the basis of the classification tasks listed in Table 1, we designed a *Rapid Miner* (RM) *workflow* (WF) [37]. RM is a software environment for rapid prototyping of machine learning and knowledge discovery (KD) processes. It is currently used for classification, clustering, and also data integration tasks, c.f.r., [38]. RM is modeled by a complex nested chain of objects called *operators*. These operators implement several KD processes, like data pre-processing, performance evaluation, learning algorithms, etc. The user is supported with graphical interfaces, where operators can be dropped as nodes onto the working pane and the data-flow is specified by connecting the operator nodes. In other words, RM workflows represent conceptual sequences of operational steps used for specific data mining experiments. Figure 6 shows the RM workflow designed for our evaluation and inference procedures. Basically, it implements standard Support Vector Machine (SVM) algorithms to forecast the patient stage. SVMs are used as “black box” inference processes to score each input dataset according to the inference performance of the algorithm [39].

The main components of the WF encode the following processes, expressed as “RapidMiner operators” are: 

• *Parameter optimization operator*. Often different learning models have many parameters and it is not clear which values are best for the learning task at hand. In order to perform the best and homogeneously as possible we optimized the AUC index over a space of given SVM feasible learning parameters. Thus, for each input, the best SVM learning parameters are found over the same space of values. The Parameter Optimization operator allows us to iteratively cycle its nested operators and change their parameters to optimize the performance of the learning scheme. In our case, the nested operator is a cross-validation process, which in turn trains and tests the SVM algorithm. In other words, we used this technique to find the best parameter combination for the SVM learning process.

• *Cross-validation operator*. This operator encapsulates a 10-fold cross-validation process. Cross-validation is a two-step process: in the first step a classifier is built describing a predetermined set of data classes. In the second step, the model (a trained SVM) is used for testing new classification examples; the generalization performance of the classifier is estimated using a new test set. The input data set *S* is split into subsets {*S*_1_,*S*_2_,…,*S*_*k*_} - in our case *k*=10. The first inner operator (SVM) realizes the learning step described above. SVM is applied 10 times using at each iteration i the set *S*_*i*_ as the test set and *S*-*S*_*i*_ as the training set. The second inner operator (model applier) realizes the second step described above. The predictive accuracy (and the other performance measures) of the classifier are then estimated using the performance operator.

In this analysis we used the following (expression level) datasets: 

• GSE27854: previously described in Section *Materials and methods*, Subsection *Gene expression analysis*.

• GSE17536: ibid.

• GSE14333: Expression values from Affymetrix Human Genome U133 Plus 2.0 array were calculated using robust multi-array average (RMA) [29]. Three groups of patients were selected: 94 patients with stage II, 91 patients with stage III, and 61 with stage IV.

From these datasets, we obtained the following *datatypes*^a^, according to the analysis provided in the previous paragraphs. 

• *Standard data* (referred to as *SA datatype*): from each dataset, the expression levels of selected up/down-regulated genes (provided through the gene expression analysis) are considered.

• *Combined data* (referred to as *COMB datatype*): from each dataset, the expression levels of selected up-regulated genes with amplification and down-regulated genes with deletion (provided through the combined gene expression and CNA analysis) are considered.

• *Relational data* (referred to as *RA datatype*): from each dataset, the dissimilarities (provided through the dissimilarity representation) between the expression levels of both the up-regulated genes with amplification and the down-regulated genes with deletion are considered.

In order to evaluate the inference performance of each datatype (thus providing an evaluation of the tumor progression inference when different information are used), we finally applied the RM-WF as reported above.

### Statistical evaluation

**Figure 7:**
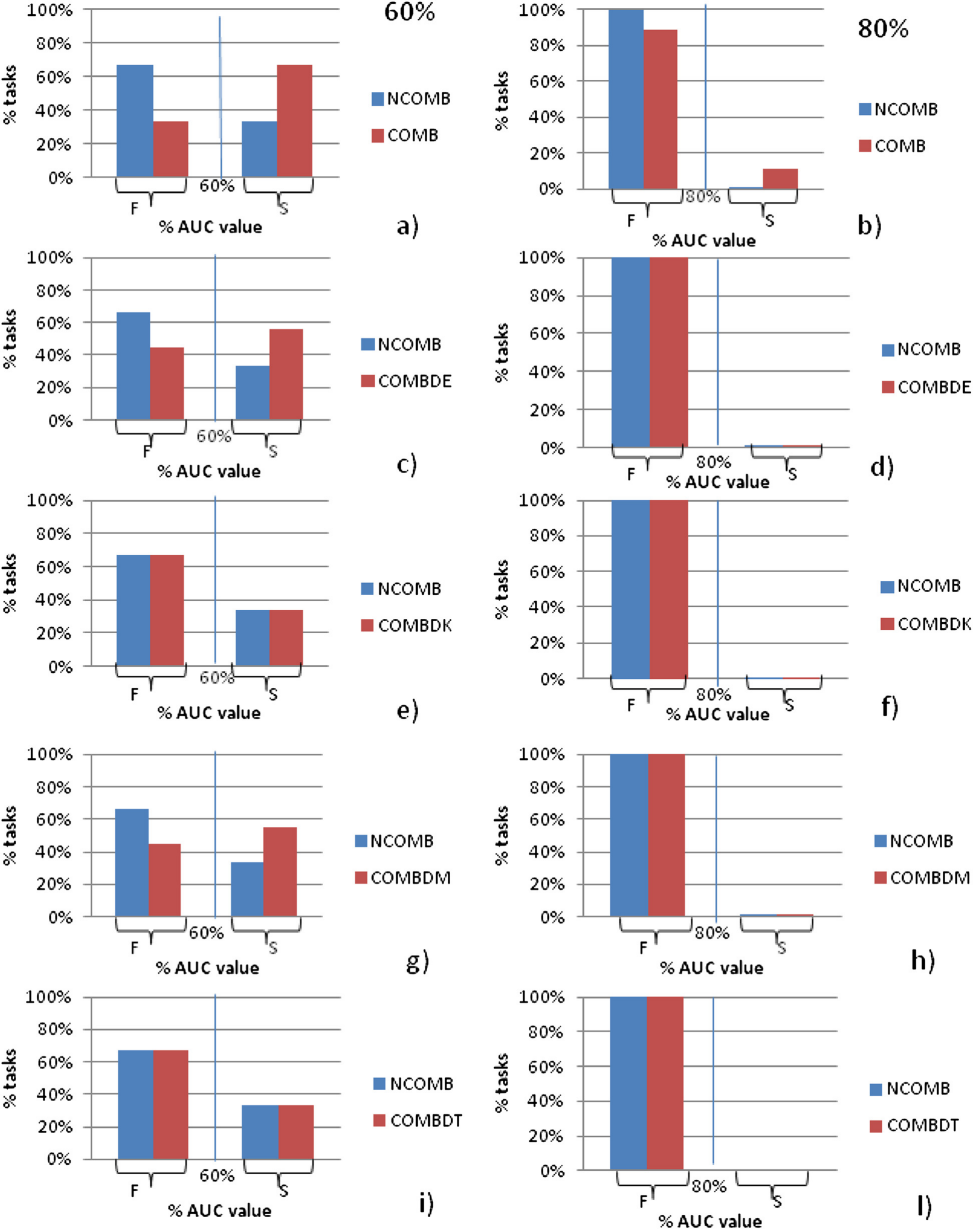
**Statistical evaluations.** Two sets: *observed set S* (success) and *set F* (failure) of AUC values according to cut off points (60% and 80% of tasks). **a)** cut off 60% for NCOMB and COMB **c)** cut off 60% for NCOMB and COMBDE **e)** cut off 60% for NCOMB and COMBDK **g)** cut off 60% for NCOMB and COMBDM **i)** cut off 60% for NCOMB and COMBDT **b)** cut off 80% for NCOMB and COMB **d)** cut off 80% for NCOMB and COMBDE **f)** cut off 80% for NCOMB and COMBDK **h)** cut off 80% for NCOMB and COMBDM **l)** cut off 80% for NCOMB and COMBDT.

In order to statistically evaluate the results of combined and/or relational information for this application we divided AUC values according to cutoff points (60% and 80%). We then evaluated two sets: 

• set *S0*: observed successes (AUC value >60*%* and AUC value >80*%*), and

• set *F0*: observed failures (AUC value ≤60*%* and AUC value ≤80*%*), as reported in Figure 7.

We then defined other two sets: 

• set *Se*: expected successes (AUC value ≥75*%*), and

• set *Fe*: expected failure (AUC value <25*%*)

We compared observed (*S0* and *F0*) and expected (*Se* and *Fe*) frequencies with the *χ*^2^ “Goodness of Fit” test, in order to answer the question whether two models (e.g., COMB and NOCOMB) are different with respect to a successes/failures composition with a defined probability of success (75%) or failures (25%).

We finally computed the residuals for each comparison criteria (|*Se*-*S*0|, |*Fe*-*F*0|).

## Ethical approval

This study was approved by the institutional review board of the Fondazione IRCCS Istituto Nazionale dei Tumori of Milan, Italy, and each patient provided written informed consent to donate the tissues left over after diagnostic procedures.

## Results

### Gene expression analysis

We found a list of up and down-regulated genes as reported in Section *Materials and methods*. This set of genes can be summarized as follows. 

• 310 up-regulated genes and 247 down-regulated genes were identified by comparing CRC data of patients with stage 2 and patients with stage 3.

• 209 up-regulated genes and 222 down-regulated genes were identified by comparing CRC data of patients with stage 2 and patients with stage 4.

• 142 up-regulated genes and 177 down-regulated genes were identified by comparing CRC data of patients with stage 3 and patients with stage 4.

### Copy number analysis

Copy number gains were frequently observed on chromosome arms 7, 8 q, 12, 13 q, and 20, copy number losses were frequently observed on chromosome arms 1 p, 5 q, 8 p, 9 q, 10 p, 14 q, 15 q, 16 p, 17, 18, 19, 20 p, and 22 q. Our findings were consistent with those published in the cytogenetic literatures [6]. These include regions frequently altered during the CRC progression.

### Combination of gene expression and genome copy number alteration

**Figure 8:**
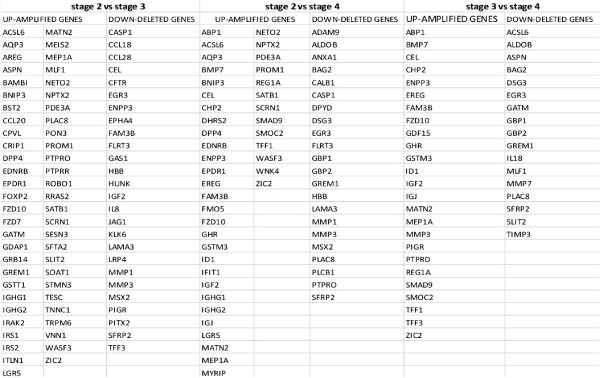
**Selected genes.** Up-Amplified and down-deleted genes for each classification task.

Up/down-regulated genes with CNAs were selected as reported in Section *Materials and methods*. Specifically, we found the genes reported in Figure 8. Here we can summarize these genes as follows. 

• 55 up-regulated genes with CNA gains were selected for the stage-2-vs-stage-3 classification task.

• 26 down-regulated genes with CNA losses were selected for the stage-2-vs-stage 3 classification task.

• 41 up-regulated genes with CNA gains were selected for the stage 2-vs-stage-4 classification task.

• 22 down-regulated genes with CNA losses were selected for the stage-2-vs-stage-4 classification task.

• 25 up-regulated genes with CNA gains were selected for the stage-3-vs-stage-4 classification task.

• 17 down-regulated genes with CNA losses were selected for the stage-3-vs-stage-4 classification task.

### Classification performances

As previously mentioned, the main issue of our investigation is to check whether the CRC progression inference benefits when considering (I) the expression levels of altered genes, and/or (II) dissimilarities between patients due to differences in the expression levels of altered genes. Here we provide cases where the performances improves by using the above information. We report the results of a comparison by employing the different datatypes reported in Section *Materials and methods*. Specifically for each task (as defined in Table 1), we verify on each dataset whether a performance improvement (with reference to the considered expression level-based information, i.e., “standard”) occurs when applying the combined and/or the relational datatypes reported in Subsection *Inference procedure and validation datasets*. In this paper, by “applying a datatype to a specific dataset” we mean that a particular information is considered (provided) from that considered dataset, e.g., consistently with the different datatype definitions, we say that the application of COMB to GSE14333 produces the expression levels of selected up-regulated genes with amplification and down-regulated genes with deletion.

All numerical experiments are evaluated by widely used indexes, mainly the AUC, to measure the capability of an inference system to classify patients.

This evaluation can be afforded, for instance, by detecting differences among a set of responses for each pair of variables *DatasetD* and *TaskT*, thus observing performances over an homogeneous source of information. Specifically, let

D={GSE14333,GSE17536,GSE27854}andT={1,2,3}

respectively the sets of all datasets and tasks considered for the inference in this work. Our evaluation is obtained by observing different performances for each pair (*d*,*t*)∈*D*×*T*, which in turn characterizes the value assumed by a new *block* variable (say, *DataTask*) when a *factor* variable (say *Criterion*) is applied to that specific dataset and task. This factor variable can take different *levels* (i.e., “treatments”) as reported in Table 2. Please refer to Section *Materials and methods* for the meaning of SA, COMB and RA datatypes.

**Table 2:** **Levels for the *factor criteria***

**Criterion**	**Applied treatment**
NCOMB	Given a task and a dataset, SA datatype is applied;
COMB	Given a task and a dataset, CA datatype is applied;
COMBED	Given a task and a dataset, RA datatype with Euclidean
	distance is applied;
COMBMD	Given a task and a dataset, RA datatype with Manhattan
	distance is applied;
COMBKD	Given a task and a dataset, RA datatype with Kullback
	distance is applied;
COMBTD	Given a task and a dataset, RA datatype with Tao
	distance is applied;

This experimental design uses a dataset for which a sample is shown in Table 3.

**Table 3:** Criteria are applied to GSE14333

**Criterion**	**DATA-task**	**AUC**
COMB	GSE14333-2VS3	0.53
NCOMB	GSE14333-2VS3	0.62
COMB	GSE14333-2VS4	0.48
NCOMB	GSE14333-2VS4	0.40
COMB	GSE14333-3VS4	0.52
NCOMB	GSE14333-3VS4	0.55
COMBDE	GSE14333-2VS3	0.63
COMBDM	GSE14333-2VS3	0.61
COMBDK	GSE14333-2VS3	0.49
COMBDT	GSE14333-2VS3	0.56
COMBDE	GSE14333-2VS4	0.51
COMBDM	GSE14333-2VS4	0.48
COMBDK	GSE14333-2VS4	0.52
COMBDT	GSE14333-2VS4	0.48
COMBDE	GSE14333-3VS4	0.55
COMBDM	GSE14333-3VS4	0.51
COMBDK	GSE14333-3VS4	0.54
COMBDT	GSE14333-3VS4	0.51

The sample size of each classification is given in Table 4. When some criterion is applied to a dataset the sample size of controls and cases are given by the associated cell reporting control groups and case groups’ size. For example, applying COMB to GSE14333 given the task 1 we have, respectively 94 controls vs. 91 cases.

**Table 4:** Sample size for each classification

**Dataset**	**Task**	***Sample size for controls***	**VS**	***Sample size for cases***
GSE14333	*Task 1*	94 (stage II)		91 (stage III)
GSE14333	*Task 2*	94 (stage II)		61 (stage IV)
GSE14333	*Task 3*	91 (stage III)		61 (stage IV)
GSE17536	*Task 1*	57 (stage II)		57 (stage III)
GSE17536	*Task 2*	57 (stage II)		39 (stage IV)
GSE17536	*Task 3*	57 (stage III)		39 (stage IV)
GSE27854	*Task 1*	41 (stage II)		35 (stage III)
GSE27854	*Task 2*	41 (stage II)		23 (stage IV)
GSE27854	*Task 3*	35 (stage III)		23 (stage IV)

When some criterion is applied the sample size for controls and cases is given by the associated cell reporting the control group’s size and case group’s size.

**Figure 9:**
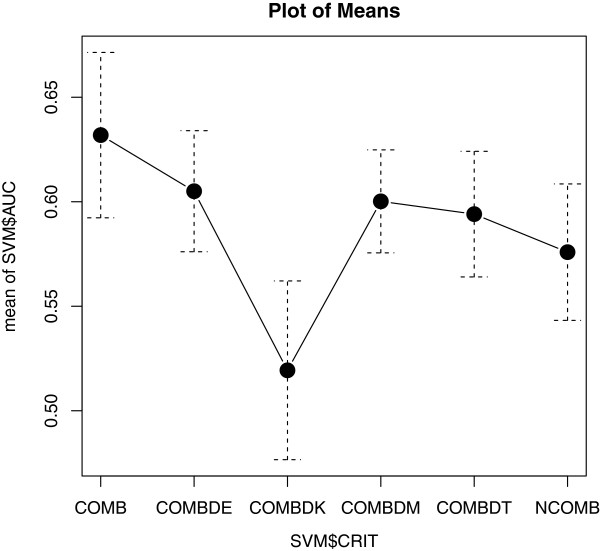
**Inference performances.** Plot of AUC mean value by Criterion. Error bars around means give plus or minus one standard error of the mean.

Our approach is empirical: we first check the discrimination performances provided by a typical standard datatype (SA-based). Then we verify whether the combined datatype (COMB-based) and/or relational datatype (RA-based) performances are able to increase the obtained SA-based performances. To give an overall judgment, reporting the Criteria which performs the best over different observations, we plots the mean performance values grouped by the factor variable Criterion. We summarize these results in Figures 9.

Criteria and Performances are reported, respectively on the *x* and *y*-axes. In these figures, we compare the observed response variables (i.e. performances by Criterion) when the RM-WF in Figure 6 is applied. Specifically, the following RapidMiner learning parameters are used: 

• kernel.type = linear;

• kernel.C.Min = -10;

• kernel.C.Max = 10000;

• kernel.C.Step = 1100

(cfr., Rapid Miner documentation [40]). We point out that performances are obtained by optimizing the AUC index over a space of common combinations of suitable SVM learning parameters, offering to the learning process the way to perform the best and homogeneously as possible for each considered DataTask input. Please note that, following this optimization we get the best SVM among a set of 1101 evaluated models (again, see [40]), i.e., each model being trained through a fixed combination of parameters given as input to the SVM learning process.

Given these premises, by considering the optimized variable AUC, we have that both COMB and 2 of the 4 considered distances (applied to COMB) improve the performance (COMBDE and COMBDM). AUC (Figure 9) is plotted vs criteria (means and standard errors represent measurements of AUC over different datasets) supporting this conclusion.

### Statistical evaluation

Figure 7(a) indicates (cut off point 60%) that 66.67*%* of tasks have AUC value greater than 60% for COMB vs 33.33*%* for NCOMB. Figure 7b) shows (cut-off point 80%) that 10% of tasks have AUC value greater than 80% for COMB, while no tasks for NCOMB achieve AUC >80*%*. Figures 7(c) and (g) (cut-off 60%) show that both COMBDE and COMBDM improve AUC performance vs NCOMB, Figures 7(e)-(f) and (i)-(e) show that COMBDK and COMBDT have similar performance to NCOMB.

Table 5 shows the *p*-value for *χ*^2^ tests for each comparison. *p*-values are all significant (≤0.001).

**Table 5:** ***p*-value of *χ***^**2 **^**test for each comparison**

	***p*-value**
COMB-NCOMB	1.1 *e*^-11^
NCOMB-COMBDE	9.6 *e*^-14^
NCOMB-COMBDK	2.5 *e*^-15^
NCOMB-COMBDM	9.6 *e*^-14^
NCOMB-COMBDT	2.5 *e*^-15^

Table 6 shows the residual. The low residual was obtained by the COMB method (both cut-off 60% and 80%) followed by COMBDE and COMBDM.

**Table 6:** **Residual for each comparison criteria (e.g., COMB(|** ***S*** ***e *- *S*** **0|, |** ***F*** ***e *- *F*** **0|) NCOMB (|** ***S*** ***e *- *S*** **0|, |** ***F*** ***e *- *F*** **0|)**

	**Cut-off**	**60%**	**80%**
COMB-NCOMB		(1;1)(4;4)	(6;6)(7;7)
NCOMB-COMBDE		(4;4)(2;2)	(7;7)(7;7)
NCOMB-COMBDK		(4;4)(4;4)	(7;7)(7;7)
NCOMB-COMBDM		(4;4)(2;2)	(7;7)(7;7)
NCOMB-COMBDT		(4;4)(4;4)	(7;7)(7;7)

*S0* and *F0* represent observed success and failure, respectively. *Se* (expected success) and *Fe* (expected failure) represent successes of expected ≥ 75% and < 25%, respectively.

## Conclusions

Previous studies integrating gene expression and copy number data have shown that changes in gene expression level between normal and tumor tissue can be associated with, and presumably caused by, changes in copy number of contiguous genes along large chromosome segments. In this paper, we showed that a prediction/classification analysis based on standard *progression stages* can be improved by using CNA-based information and/or dissimilarity representation of patients. RA and/or COMB, thanks to the chosen distances (and data), allowed SVMs to outperform (on the given inference tasks) a typical *standard representation* approach, where patients are categorized by their set of available attribute values.

To summarize, the following simple pipeline for the CRC progression inference can be used. 

1. Differentially expressed genes are selected by evaluating their expression levels on different datasets.

2. Similarly, altered genes are located.

3. Differentially expressed genes with CNAs are identified.

4. Disease progression inferences based on the classification tasks reported in Table 1 can be obtained by applying the Rapid Miner workflow in Figure 6. This workflow and a sample dataset are available for download at http://bimib.disco.unimib.it/index.php/Publications/JCBI/.

We point out that the optimization procedure in Figure 6 is based around the search for the best performing model in such a way that SVMs (i.e., trained models) work the best for all applied datatypes. In other words, here we enforced the search for an accurate system which, at the best of its ability, could eventually benefit when using combined and/or relational data. Clearly, in order to give significant evidence of the usefulness of combined and/or relational information for this application, more datasets and models have to be compared through suitable statistical tests, with the goal to take into account the not-so-straightforward applicability of the required statistical assumptions for the machine learning algorithms; see for instance the recent book [41]. This is a first extension to this work, which we are immediately interested for our future analyses.

Defining a well-discriminating dissimilarity function, in this framework, is difficult. In this work, our choice was to apply standard metrics. Differently to SA, “dissimilarities” focus on group or subject differences. Indeed, we first defined *prototype* patients. Then we represented case/control patients through their set of distances from the considered prototype instances. Finally, we based the inference on different discrimination tasks, i.e., using a *case vs. control* “design” between groups.

The choice of a correct prototype set can be critical in this approach. This is another question which we are immediately interested in a future study. We did not study the best possible prototype set, instead we used the group with the lowest available progression’s marker.

Finally, other interesting extensions could be provided by integrating different CNA-based information, for instance concerning chromosome specific regions or the *probe* number used for each aberrant region.

Many genes selected in our analyses (see Figure 8) were already identified either as oncogenes or transcription factors (some of them promote tumor growth and proliferation) according to *CANCER GENES*[42] and *CGAP*[43].

Table 7 shows up-amplified genes and their functions: i) up-amplified genes selected both for the stage-2-vs-3 and stage-2-vs-4 classification, ii) up-amplified genes for the stage-2-vs-stage-3 classification iii) up-amplified genes for stage-3-vs-stage-4.

**Table 7:** Up-amplified genes

**Stage2 vs stage3 and stage2 vs stage4**	
**Gene**	**Function**
SATB1	*promotes the cell growth*
	*and reduces apoptosis*
BNIP3	*is involved in mTOR signaling*
	*(resulting in increased*
	*protein translation)*
EDNRB	*(a transactivator of EGFR)*
	*induces tumor growth*
AQP3	*facilitate colorectal carcinoma*
	*cell migration *[44]
LGR5	*Its expression is significantly*
	*higher in carcinoma than in*
	*normal mucosa *[45]
SCRN1	*associate to a poor prognosis *[46]
**Stage2 vs stage3**	
**Gene**	**Function**
AREG and GRB14	*promote proliferation and*
	*interact with EGFR*
BAMBI	*It is involved in TGF-beta*
	*receptor signaling pathway*
	*(growth induction),*
FZD7	*participates to the WNT*
	*signaling pathway*
IRS1 and IRS2	*They are activated by insulin*
PTPRR	*It is activated from the MAPK*
	*signaling pathway*
**Stage3 vs stage4**	
**Gene**	**Function**
EREG	*which promotes proliferation*
	*and interacts with EGFR*
IGF2	*It is involved in TGF-beta*
	*receptor signaling pathway*
	*(growth induction),*
TFF1 and TTF3	*the growth factors*
BMP7 and SMAD9	*involved in BMP receptor*
	*signaling genes involved in BMP*
	*receptor signaling*
GDF15 and ID1	*growth factors involved in the*
	*TGF-beta signaling pathway*

Table 8 shows down-deleted genes and their functions: *i*) down-deleted genes selected both for the stage-2-vs-3 and stage-2-vs-4 classification, *ii*) down-deleted genes for the stage-2-vs-stage-3 classification *iii*) down deleted genes for stage-3-vs-stage-4. The above gene selection (in agreement with the identified oncogenes or transcription factors) is a result supporting the relevance of gained and lost regions for cancer progression as useful signals to distinguish the different considered classes.

**Table 8:** Down-deleted genes

**Stage2 vs stage3 and stage2 vs stage4**	
**Gene**	**Function**
CASP1 and LAMA3	*regulate cell adhesion*
MSX2	*blocks cell proliferation)*
SFRP2	*(is a tumor suppressor gene*
	*frequently methylated in CRC)*
**Stage2 vs stage3**	
**Gene**	**Function**
GAS1 and KLK6, FAM3B	*induce apoptosis*
LRP4	*a negative regulator of WNT*
	*signaling pathway*
PITX2	*is a regulator of beta-catenin*
	*signaling*
**Stage3 vs stage4**	
**Gene**	**Function**
SLIT2	*is a positive regulator of*
	*apoptosis and blocks migration*
TIMP3	*is involved in p53*
	*signaling pathway,*
MLF1	*induces cell cycle arrest*

## Endnote

^a^ We use the term datatype to generalize the specific data representation under analysis.

## Competing interests

The authors declare that they have no competing interests.

## Authors’ contributions

The overall layout of the biological and technical analysis grew from discussions between IZ and CC. CC carried out the molecular genetic studies, participated both in the numerical evaluation analysis and to draft the manuscript. IZ carried out the statistical analysis, the applied dissimilarity-based representation and participated both to the numerical evaluation analysis and to draft the manuscript. MG carried out the selected gene identification. IC supervised the work from the biological point of view and revised the manuscript. GM supervised the work from the technical point of view and participated in the project coordination. MA conceived this study in the context of the BIMIB group’s activities, suggested the dissimilarity-baed approach, coordinated the project and edited the manuscript. All authors read and approved the final manuscript.
